# 5-HEPE Ameliorates Aging of Duck Ovarian Granulosa Cells by Targeting FOXM1 and Suppressing Oxidative Stress

**DOI:** 10.3390/antiox14121425

**Published:** 2025-11-27

**Authors:** Yibo Zong, Jinyu Liu, Wenwu Xu, Tiantian Gu, Yong Tian, Li Chen, Tao Zeng, Lizhi Lu

**Affiliations:** State Key Laboratory for Quality and Safety of Agro-Products, Key Laboratory of Livestock and Poultry Resources (Poultry) Evaluation and Utilization, Ministry of Agriculture and Rural Affairs, Zhejiang Key Laboratory of Livestock and Poultry Biotech Breeding, Zhejiang Provincial Engineering Research Center for Poultry Breeding Industry and Green Farming Technology, Institute of Animal Husbandry and Veterinary Science, Zhejiang Academy of Agricultural Sciences, Hangzhou 310021, China

**Keywords:** laying duck, ovarian aging, oxylipin, oxidative stress, 5-HEPE

## Abstract

Oxylipins are tightly linked to ovarian function. However, the roles and mechanisms of oxylipins in ovarian aging of laying ducks remain poorly understood. In this study, 72-week-old laying Jinyun ducks were categorized into high-laying (HL) and low-laying (LL) groups. Plasma and ovarian tissues were analyzed for antioxidant indices, transcriptomic, and targeted lipidomic. Results demonstrated that, compared to the LL group, the HL group ducks exhibited lower levels of MDA in both plasma and ovarian tissues, while exhibiting higher levels of SOD, GSH-Px, and T-AOC. Differentially expressed genes were primarily enriched in fatty acid oxidation and inflammation-related pathways, with FOXM1 identified as a pivotal gene involved in delaying ovarian aging. Furthermore, oxylipin profiles in plasma and ovary exhibit marked distinctions between the HL and LL group ducks. Notably, 5-hydroxy-eicosapentaenoic acid (5-HEPE) was substantially upregulated in both plasma and ovaries of the HL group ducks. Meanwhile, 5-HEPE significantly enhanced FOXM1 expression and mitigated oxidative stress in granulosa cells. Collectively, this study provides the first comprehensive analysis of oxylipin changes in the ovaries and plasma of ducks with differing laying performances at the late-laying stage. These findings offer novel insights into the prevention and alleviation of ovarian oxidative stress in ducks during this period.

## 1. Introduction

The decline in ovarian function remains a primary determinant of reduced reproductive lifespan and compromised egg quality in laying ducks. The ovary, as the central organ of egg production, plays a pivotal role in follicular growth, development, and function, all of which are crucial for determining laying performance. Follicles, the fundamental functional units of the ovary, comprise oocytes and initiate their development at the onset of laying. Commercial poultry with a high egg-laying rate consistently maintain a relatively high follicular development rate [1]. Prior to laying, primordial follicles are activated within the ovarian cortex, transitioning into primary follicles, a process driven by the development of a single layer of granulosa cells (GCs), followed by the formation of multilayered follicular structures [2]. GCs serve not only as a functional hub for ovarian reproductive and endocrine activity but also as a sensitive target of oxidative stress and aging. Aging is a natural physiological and pathological phenomenon. Notably, the female reproductive system undergoes aging more rapidly than other physiological systems [3]. Thus, understanding the molecular basis of ovarian aging and identifying anti-aging regulators is critical for delaying ovarian decline and extending the laying cycle. Redox imbalance within the ovarian microenvironment is a key driver of ovarian aging [4]. Senescent cells retain metabolic activity and are able to produce and secrete a multitude of metabolites that influence the tissue microenvironment through various means [5,6]. However, how specific metabolites modulate GC function during aging remains a critical, unanswered question.

Oxylipins are metabolites generated from polyunsaturated fatty acids (PUFAs) through autoxidation or enzymatic catalysis by specific enzymes [7]. They exert important regulatory functions in immune modulation, oxidative injury, and inflammatory processes [8,9], and accumulating evidence indicates a close relationship between oxylipins and ovarian function [8,10]. Sustained, high-level egg production imposes an immense metabolic and oxidative burden on the avian ovary [11]. This high-stress environment necessitates robust adaptive mechanisms to maintain cellular homeostasis and prevent premature senescence. Oxylipins play an important role in mediating this homeostasis. 5-hydroxy-eicosapentaenoic acid (5-HEPE), formed through 5-LOX-catalyzed metabolism of eicosapentaenoic acid (EPA), has been shown to modulate metabolic and inflammatory processes [12,13]. The roles of oxylipins are characterized in mammals. However, substantial differences exist between poultry and mammalian ovaries. The influence of oxylipins on ovarian function in poultry has not yet been reported. Therefore, systematic investigation and elucidation of oxylipin effects on ovarian and GC function are essential for breeding laying ducks with extended laying cycles.

At the cellular level, senescence is marked by a pronounced reduction in proliferative capacity, which is linked to several well-established hallmarks of aging, including genomic instability, epigenetic modifications, and cellular senescence itself [14,15]. Regulation of the proliferation-associated transcription factor FOXM1 has been reported to ameliorate multiple aging phenotypes, such as the accumulation of pro-inflammatory senescent cells [16]. Nevertheless, owing to the pleiotropic functions of FOXM1 across diverse biological pathways, its role in GCs of the avian ovary, particularly during the late laying stage, remains inadequately characterized.

In this study, 72-week-old Jinyun ducks were selected and stratified into HL and LL groups according to egg production. Targeted lipidomics was employed to quantify oxylipins in plasma and ovarian tissues, while ovarian transcriptome sequencing was integrated to identify aging-related genes. The objective was to elucidate the potential molecular mechanisms by which oxylipins regulate ovarian aging within the ovarian microenvironment of laying ducks.

## 2. Materials and Methods

### 2.1. Animal Experiments and Sample Collection

Twenty-four 72-week-old laying Jinyun ducks (Anas platyrhynchos) were purchased from Guowei Poultry Industry Co., Ltd. (Shaoxing, China). These ducks were categorized into high-laying (HL) groups and low-laying (LL) groups according to egg production (Supplementary Materials). The ducks were reared in three-tiered caged housing (length × width × height, 28 × 40 × 40 cm), with 2 ducks per cage. Diet and water were provided ad libitum. Venous blood samples were procured through the brachial vein, followed by plasma separation. Ducks were injected with pentobarbital sodium (150 mg/kg) for anesthesia. Then, the duck’s abdominal cavity was dissected. Ovarian tissue was isolated for further experimental analyses.

### 2.2. Cell Culture

The immortalized human granulosa cell line KGN cells were acquired from Procell (Wuhan, China) and cultured in DMEM/F12 medium containing 10% fetal bovine serum and 1% penicillin–streptomycin. The cells were incubated at 37 °C with 5% CO_2_. Primary ovarian granulosa cells were isolated from Jinyun ducks. Ovaries were aseptically dissected, and 2–5 mm secondary follicles were excised and stripped of theca layers. Note that after cutting the follicles with sterile scissors, allow the yolk fluid to flow out, then rinse repeatedly with PBS to remove residual yolk. Ovarian follicles were subjected to enzymatic digestion using 0.1% collagenase IV and 0.05% hyaluronidase in DMEM/F12 medium. The resulting suspension was then filtered through a 75-μm mesh to remove cellular debris, and GCs were pelleted via centrifugation at 1000× *g* for 5 min. After centrifugation, the cells were resuspended in complete DMEM/F12 medium containing 10% fetal bovine serum and 1% penicillin–streptomycin and subsequently plated into culture dishes. All cultures were maintained at 37 °C in a 5% CO_2_ atmosphere. 5-HEPE was acquired from Cayman Chemical (Ann Arbor, MI, USA). 5-HEPE is finally dissolved in PBS [13] and then added to the culture medium at the required dosage to treat the cells.

### 2.3. Determination of Oxidative Stress Indicators Content

The concentrations of malondialdehyde (MDA; BC0020), total antioxidant capacity (T-AOC; BC1310), superoxide dismutase (SOD; BC0170), and glutathione peroxidase (GSH-Px; BC1190) in plasma, ovarian tissues, and cellular samples were assessed using corresponding commercial assay kits (Solarbio, Beijing, China) in accordance with the manufacturer’s protocols. The value was obtained by a microplate reader.

### 2.4. Hematoxylin and Eosin Staining

After fixation in paraformaldehyde, the ovaries were embedded in paraffin wax and then processed into tissue sections. The sections were dewaxed in xylene and rehydrated through a graded ethanol series, then stained by hematoxylin, differentiated in hydrochloric acid-ethanol, and counterstained with eosin. Following gradient ethanol dehydration and xylene clearing, sections were finally sealed using the sealing agent. Images were captured under a microscope (DM6000B).

### 2.5. Cell Viability Assay

KGN cells were grown to 80% in 96-well plates, and treated with hydrogen peroxide at different concentrations for 2 h, and their viability was measured in accordance with the instructions provided in the CCK-8 kit (Beyotime, Shanghai, China).

### 2.6. mRNA Extraction and Quantitative Real-Time PCR (qRT-PCR)

Trizol (APPLYGEN, Beijing, China) was used to extract RNA from tissues or cells, following the manufacturer’s protocol. Briefly, chloroform was added to separate the mixture into aqueous and organic phases. The RNA-containing aqueous phase was carefully transferred, and RNA was precipitated using isopropanol. The resulting precipitates underwent washing with 75% ethanol, were air-dried, and then reconstituted in nuclease-free H_2_O. RNA concentration and purity were assessed using a spectrophotometer. cDNA synthesis was performed with a reverse transcription kit (TransGen Biotech, Beijing, China). qRT-PCR was conducted with the application of a SYBR Green master mix, utilizing a real-time PCR determination platform. Gene expression levels were normalized against the expression of β-actin. The sequences of the primers utilized in this study are provided in Table 1.

### 2.7. Protein Extraction and Western Blot Analysis

Ovarian tissues and cells were homogenized in ice-cold RIPA lysis buffer containing protease inhibitors. Protein concentrations were quantified with a BCA assay kit [17]. Subsequently, protein specimens were combined with 5× SDS buffer and subjected to denaturation at 95 °C for 7 min. Following denaturation, the proteins were resolved using SDS-PAGE and underwent electrophoretic transfer to a PVDF membrane. After transfer, the membranes were treated with a rapid blocking solution (Servicebio, Wuhan, China) for 5 min at room temperature. Blots were then probed with primary antibodies against FOXM1 (ab245309, Abcam (Cambridge, UK)), GPX4 (3038-1-AP, Proteintech (Rosemont, IL, USA)) and β-actin (10536-1-AP, Proteintech) at 4 °C. Following primary antibody incubation, the membrane was incubated with a secondary antibody conjugated to HRP for 1 h at room temperature. Protein bands were detected via a chemiluminescence imaging system and subjected to quantitative analysis using ImageJ software (2.3.0 Rasband, W.S., ImageJ, U. S. National Institutes of Health, Bethesda, MD, USA, https://imagej.net/ij/, accessed on 11 September 2025), with β-actin serving as the internal loading control.

### 2.8. RNA Sequencing

Ovarian tissue samples were collected from laying ducks (*n* = 5), and ovarian tissue total RNA was extracted using TRIzol reagent (Thermo Scientific, Waltham, MA, USA). The purity, integrity, and concentration were validated via Nanodrop spectrophotometry, Agilent 2100 Bioanalyzer (Santa Clara, CA, USA), and agarose gel electrophoresis, respectively. For cDNA library construction, high-quality RNA was processed following the manufacturer’s protocol of the Illumina TruSeq RNA Sample Prep Kit: ribosomal RNA (rRNA) was first depleted, followed by reverse transcription to synthesize cDNA. The resulting cDNA underwent end repair, A-tailing, and adapter ligation, and was then amplified by PCR and subjected to fragment size selection. After quality verification of the libraries, paired-end sequencing was performed on an Illumina NovaSeq 6000 platform. Raw sequencing data were filtered to remove low-quality reads and adapter sequences, yielding clean reads for subsequent bioinformatic analyses. Differential expression analysis was performed using the DESeq25. Q value < 0.05 and foldchange > 2 or foldchange < 0.5 was set as the threshold for significantly differential expression gene (DEGs).

### 2.9. Targeted Lipidomics

Ovarian tissue and plasma samples were collected from laying ducks (*n* = 4). All eicosanoids and deuterium-labeled internal standards were acquired from Cayman Chemical. Each 20 mg sample was combined with 200 μL of a methanol/acetonitrile (1:1, *v*/*v*) solution spiked with internal standards. Tissue disruption was performed using a homogenizer at 30 Hz for 20 s. To precipitate proteins, the mixtures were kept at −20 °C for 30 min and then centrifuged at 11,000× *g* and 4 °C for 10 min. The supernatant was harvested, and the extraction process was repeated. Pooled supernatants were subjected to solid-phase extraction on Poly-Sery MAX SPE columns (ANPEL). The eluate was dried under vacuum and redissolved in 100 μL of methanol/water (1:1, *v*/*v*) prior to instrumental analysis. Quantification of oxylipins was carried out using the MetWare platform (http://www.metware.cn/, accessed on 20 August 2025) based on an AB Sciex QTRAP 6500 LC-MS/MS system.

### 2.10. Statistical Analysis

Data are expressed as the mean ± standard error of the mean (SEM). Statistical analysis was conducted using one-way ANOVA or Student’s *t*-test. A *p*-value of <0.05 was considered statistically significant.

## 3. Results

### 3.1. Ovarian Morphology of Laying Ducks with Different Egg Production Levels in the Late Laying Period

**Figure 1:**
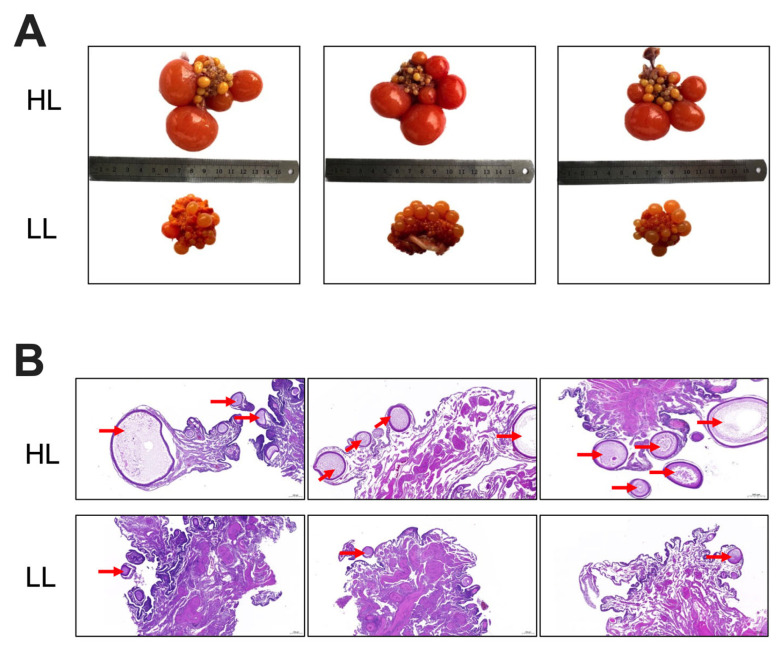
Ovarian morphology of laying ducks with different egg production levels in the late laying period. (**A**) Representative photographs of the ovary, the red arrows indicate the follicles. (**B**) HE staining of the ovarian sections (*n* = 6).

Overall picture revealed distinct differences in ovarian morphology between the two groups, with the HL group ducks’ ovary exhibiting relatively balanced follicular development across all stages (Figure 1A). H&E staining showed that ovarian connective tissue in the HL group ducks contained healthy, intact follicles at all stages with well-organized and clearly defined GC layers. In contrast, the LL group duck ovary displayed denser ovarian connective tissue, a greater number of atretic follicles, and severe disruption of granulosa layer structures (Figure 1B).

### 3.2. Transcriptome Analysis in the Ovary of Laying Ducks

**Figure 2:**
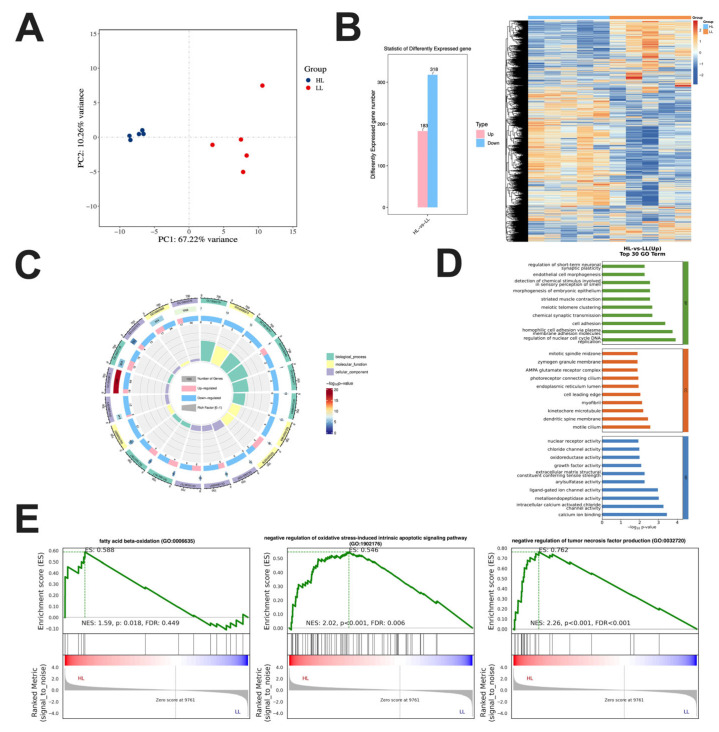
Transcriptome analysis in the ovary of laying ducks. (**A**) PCA. (**B**) Cluster heatmap. (**C**) Enrichment analysis loop diagram. (**D**) GO enrichment. (**E**) GSEA enrichment map (*n* = 5).

To explore the molecular mechanisms of ovarian aging during the late laying stage, RNA-seq was performed on ovarian tissues from HL and LL ducks. Principal component analysis (PCA) demonstrated clear separation between the two groups along the first two principal components, indicating marked differences in gene expression profiles (Figure 2A). A heatmap of differentially expressed genes (DEGs) identified 501 DEGs, including 183 upregulated and 318 downregulated genes (Figure 2B). Circos plots and Gene Ontology (GO) enrichment analyses revealed that these DEGs were predominantly associated with pathways related to lipid metabolism, oxidative stress responses, and inflammatory regulation (Figure 2C,D). Gene set enrichment analysis (GSEA) further indicated that DEGs in the HL group were primarily involved in regulating fatty acid oxidation, as well as suppressing oxidative stress responses and negatively regulating the production of pro-inflammatory mediators (Figure 2E).

### 3.3. FOXM1 Was Upregulated in the Duck Ovary of the HL Group

**Figure 3:**
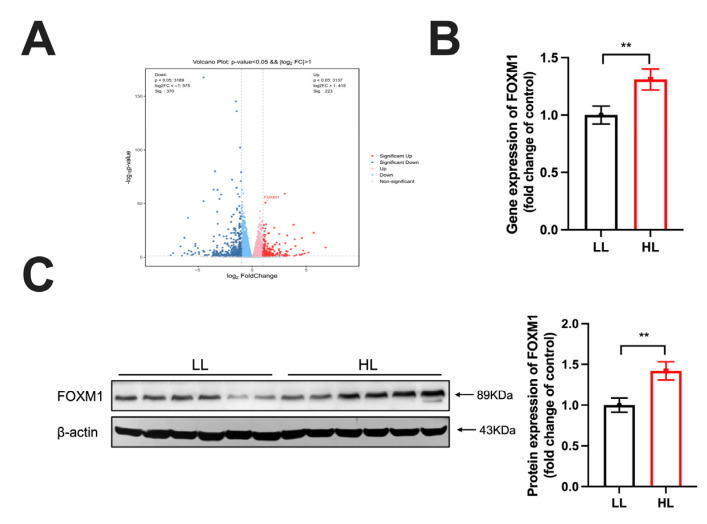
FOXM1 is upregulated in the duck ovaries of the HL group. (**A**) Volcano plot (**B**,**C**) Gene and protein expression of FOXM1 in the ovary (*n* = 6). Data are presented as the mean ± SEM, with statistical significance denoted as ** *p* < 0.01.

Volcano plots revealed elevated FOXM1 expression in HL ovaries (Figure 3A). FOXM1 has been recognized as a pivotal anti-aging regulator [18]. Consistently, both mRNA and protein expression of FOXM1 in the ovaries of HL-group laying ducks were significantly higher than that in LL-group laying ducks (Figure 3B,C).

### 3.4. Differences in Oxidative Stress Between the HL and LL Group Ducks

**Figure 4:**
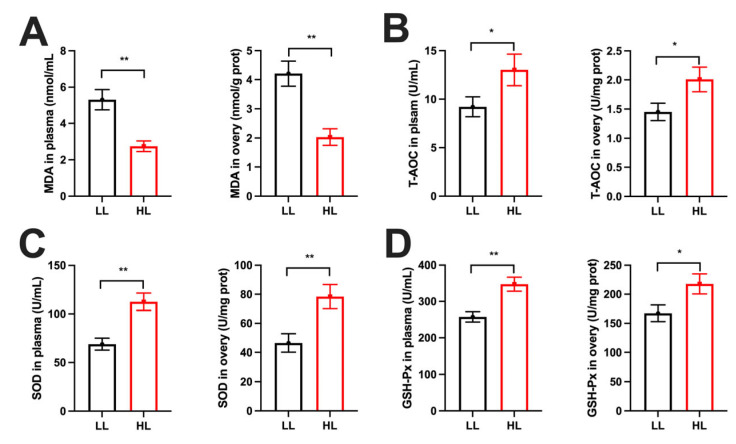
Differences in oxidative stress between the HL and LL group ducks. (**A**) MDA, (**B**) T-AOC, (**C**) SOD, and (**D**) GSH-Px levels in plasma and ovaries (*n* = 8). Data are presented as the mean ± SEM, with statistical significance denoted as * *p* < 0.05, ** *p* < 0.01.

To assess oxidative stress status in late-laying ducks, oxidative indices in plasma and ovarian tissues were measured. MDA levels were significantly lower in both plasma and ovaries of the HL group ducks compared with the LL group (Figure 4A). Conversely, T-AOC (Figure 4B), SOD (Figure 4C), and GSH-Px (Figure 4D) levels were markedly higher in the HL group ducks.

### 3.5. Targeted Lipidomics Analysis in Plasma of Laying Ducks

**Figure 5:**
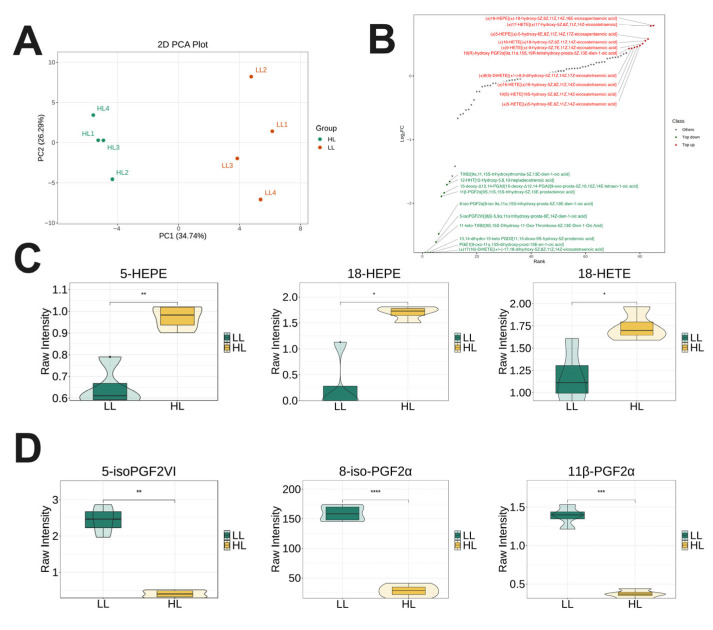
Targeted lipidomics analysis in plasma of laying ducks. (**A**) PCA. (**B**) Dynamic distribution map of oxylipin content differences. (**C**) Representative of upregulated oxylipins. (**D**) Representative of downregulated oxylipins. (*n* = 4). Data are presented as the mean ± SEM, with statistical significance denoted as * *p* < 0.05, ** *p* < 0.01, *** *p* < 0.001, **** *p* < 0.0001.

Targeted lipidomics was conducted to evaluate differences in oxylipin profiles. PCA revealed distinct separation between the HL and LL group ducks along the principal components (Figure 5A). Differential analysis identified oxylipins with significant variation, with the top 10 upregulated and downregulated metabolites annotated (Figure 5B). Specifically, plasma levels of 5-HEPE, 18-HEPE, and 11-HETE were significantly elevated in the HL group (Figure 5C), whereas levels of 5-HPETE, 8-iso-PGF2α, and 11β-PGF2α were markedly reduced (Figure 5D).

### 3.6. Targeted Lipidomics Analysis in the Ovary of Laying Ducks

**Figure 6:**
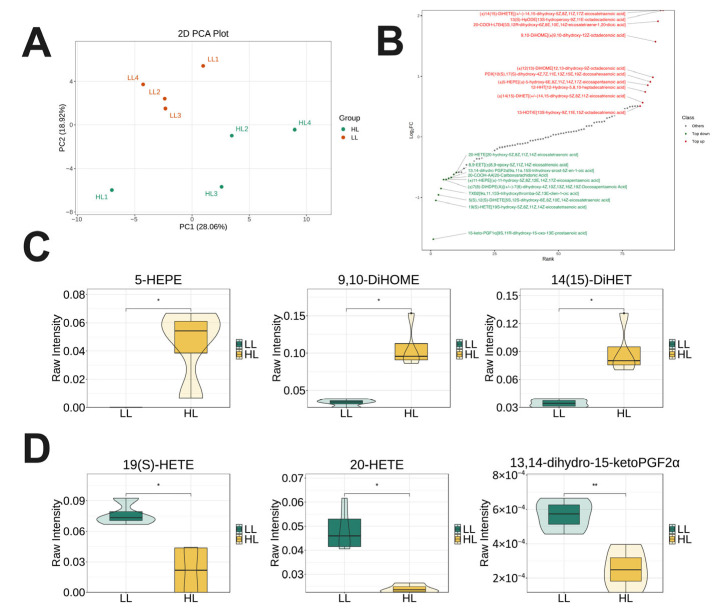
Targeted lipidomics analysis in the ovary of laying ducks. (**A**) PCA. (**B**) Dynamic distribution map of oxylipin content differences. (**C**) Representative of upregulated oxylipins. (**D**) Representative of downregulated oxylipins. (*n* = 4). Data are presented as the mean ± SEM, with statistical significance denoted as * *p* < 0.05, ** *p* < 0.01.

Targeted lipidomics of the ovary revealed distinct separation between the HL and LL groups by PCA, with strong intra-group reproducibility (Figure 6A). Differential analysis identified oxylipins with significant variation, with the top 10 upregulated and downregulated metabolites annotated (Figure 6B). The levels of 5-HEPE, 9,10-DiHOME, and 14,15-DiHETrE were significantly elevated in the HL group ovary (Figure 6C), whereas 13(S)-HETrE, 20-HETrE, and 11,12-DiHETrE levels were markedly reduced (Figure 6D).

### 3.7. 5-HEPE Alleviated Oxidative Stress and Upregulated FOXM1 Expression in KGN Cells

**Figure 7:**
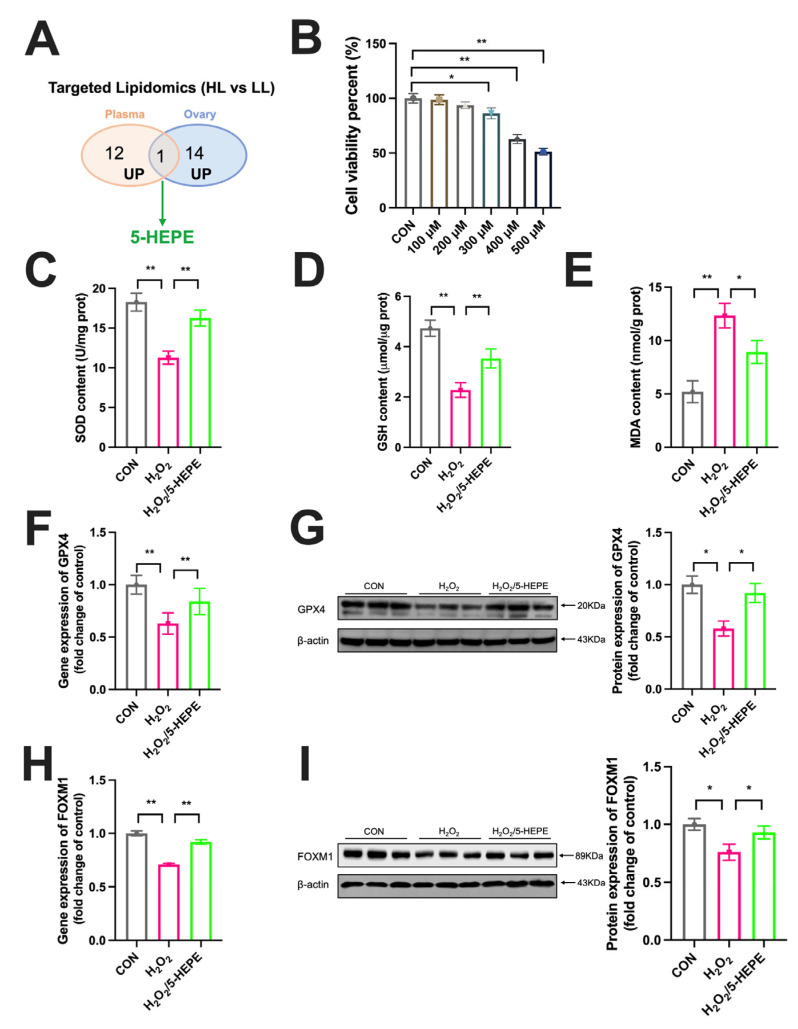
5-HEPE alleviated oxidative stress and upregulated FOXM1 expression in KGN cells. (**A**) Differential omics profile in plasma and ovary. (**B**) Cell viability of KGN cells after treatment with different concentrations of H_2_O_2_. (**C**–**E**) GSH, SOD, and MDA content in KGN cells. (**F**,**G**) Gene and protein expression of GPX4 in KGN cells. (**H**,**I**) Gene and protein expression of FOXM1 in KGN cells. (*n* = 3). Data are presented as the mean ± SEM, with statistical significance denoted as * *p* < 0.05, ** *p* < 0.01.

5-HEPE was identified as a common differential oxylipin in both plasma and ovarian lipidomes (Figure 7A). Cell viability assay demonstrated that 300 μM H_2_O_2_ markedly reduced KGN cell viability, and 400 μM H_2_O_2_ decreased KGN cell viability by approximately 45% (Figure 7B). Therefore, 400 μM H_2_O_2_ was used to establish a cellular oxidative damage model. 5-HEPE significantly mitigated the H_2_O_2_-induced decline in SOD and GSH levels and the increase in MDA levels in KGN cells (Figure 7C,E). 5-HEPE also significantly alleviated the inhibitory effect of H_2_O_2_ on the gene and protein expression of GPX4 (Figure 7F,G). Moreover, 5-HEPE markedly attenuated the inhibitory effect of H_2_O_2_ on FOXM1 expression (Figure 7H,I).

### 3.8. 5-HEPE Alleviated Oxidative Stress and Upregulated FOXM1 Expression in Ovarian GCs of Laying Ducks

**Figure 8:**
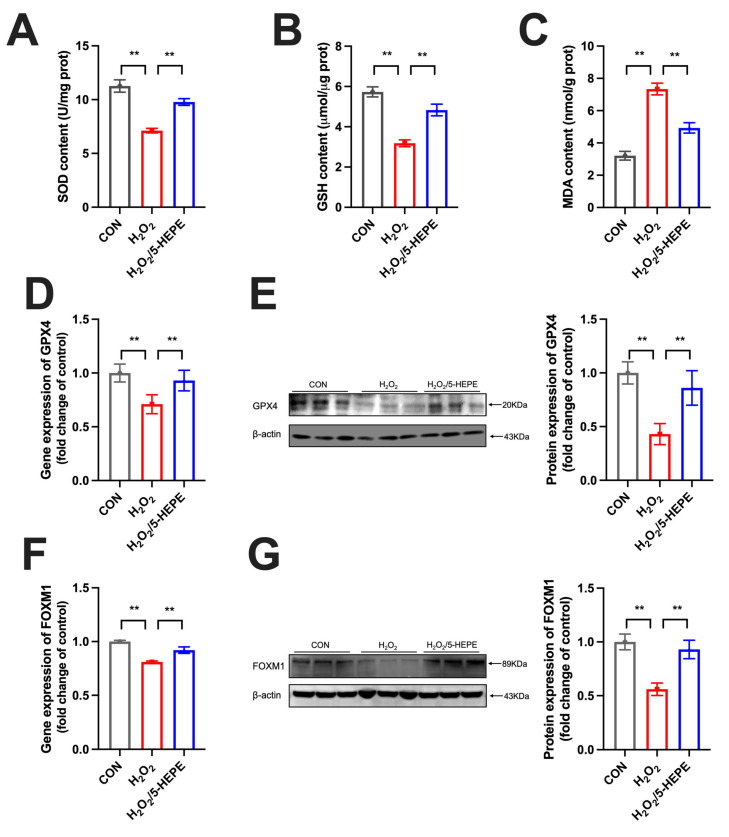
5-HEPE alleviated oxidative stress and upregulated FOXM1 expression in ovarian GCs of laying ducks. (**A**–**C**) GSH, SOD, and MDA content in laying ducks’ ovarian GCs. (**D**,**E**) Gene and protein expression of GPX4 in ovarian GCs of laying ducks. (**F**,**G**) Gene and protein expression of FOXM1in ovarian GCs of laying ducks. (*n* = 3). Data are presented as the mean ± SEM, with statistical significance denoted as ** *p* < 0.01.

Ovarian GCs were isolated from laying ducks to further examine the effect of 5-HEPE on GCs oxidative damage. Treatment with H_2_O_2_ (400 μM) significantly decreased SOD and GSH levels in GCs, while markedly increasing MDA levels. The addition of 5-HEPE (50 μM) significantly restored SOD and GSH levels and reduced MDA content (Figure 8A–C). 5-HEPE also significantly alleviated the inhibitory effect of H_2_O_2_ on the gene and protein expression of GPX4 (Figure 8D,E). Meanwhile, 5-HEPE substantially mitigated the inhibitory effect of H_2_O_2_ on FOXM1 expression (Figure 8F,G).

## 4. Discussion

Aging is a natural physiological process throughout the life cycle, characterized by progressive decline in tissue and organ function [19,20]. In avian species, ovarian health is a critical determinant of reproductive longevity and laying performance [2,21], with ovarian aging being a primary factor that curtails the laying cycle [22]. Accumulating evidence has demonstrated that oxylipins are closely associated with ovarian function [23,24]. Elucidating the complex interactions between oxylipins and ovarian tissues provides new opportunities for investigating strategies to delay ovarian aging. This study provides a comprehensive analysis of oxylipin profiles in the peripheral blood and ovaries of laying ducks with divergent egg production, identifying key regulatory molecules and genes. Oxidative stress and inflammation are major contributors to accelerated ovarian aging [25,26,27]. In this study, GSEA of the ovarian transcriptome revealed that pathways suppressing oxidative stress-induced apoptosis, promoting fatty acid oxidation, and inhibiting tumor necrosis factor signaling were significantly enriched in the high-producing (HL) ducks. These findings underscore the importance of enhanced fatty acid metabolism and robust anti-inflammatory capacity in maintaining ovarian function and sustaining high egg production during the late laying period. Our results are consistent with previous reports showing that ceased-laying ducks exhibit significantly greater oxidative damage in their ovaries compared to their laying counterparts [28]. Consistent with this, the present data showed that T-AOC, SOD, and GSH-Px levels in both plasma and ovarian tissues were significantly elevated in the HL group ducks compared with the LL group, whereas MDA concentrations in plasma and ovarian tissues were markedly reduced in the HL group ducks.

Dysregulation of oxylipin metabolism is frequently implicated in ovarian pathologies, including premature ovarian insufficiency [29,30,31]. Our investigation revealed a distinct shift in the plasma and ovarian oxylipin profiles between the HL and LL groups. 18-HEPE is recognized as a biomarker of EPA-mediated inflammation resolution and exhibits antiviral activity [32,33]. As an EPA metabolite, 5-HEPE has been shown to enhance regulatory T cell induction by adipose tissue macrophages [34]. In this study, plasma levels of both 5-HEPE and 18-HEPE were significantly higher in HL ducks than in LL ducks. Conversely, 5-isoPGF2VI, 8-iso-PGF2α, and 11β-PGF2α, all of which exert pro-inflammatory effects and promote the progression of ovarian cancer [35,36,37], were significantly lower in HL plasma. Notably, 8-iso-PGF2α also serves as a key mediator of cellular oxidative injury [38]. Consistent with plasma findings, 5-HEPE levels were significantly elevated in HL ovaries. Previous research reported that 5-HEPE can markedly upregulate NRF2 expression in vascular endothelial cells [39]. In this study, 5-HEPE significantly mitigated H_2_O_2_-induced oxidative damage in both duck ovarian GCs and KGN cells. The pivotal enzyme responsible for this conversion is 5-lipoxygenase (ALOX5), which catalyzes the oxygenation of EPA to generate 5-HEPE [40]. Although ALOX5 expression was not quantified in the current study, the substantial accumulation of its downstream product strongly implies that the ALOX5 pathway may be upregulated in the ovaries of high-producing ducks. This potential enhancement is particularly significant as the ALOX5 pathway is a cornerstone in the synthesis of specialized pro-resolving mediators (SPMs), which are crucial for actively terminating inflammation rather than merely suppressing it. Therefore, an enhanced ALOX5-mediated synthesis of 5-HEPE could be a key mechanism underpinning the pro-resolving and anti-senescence phenotype observed in HL ducks. Future studies directly investigating the expression, activity, and regulation of ALOX5 in the avian ovary are warranted to validate this hypothesis and further elucidate its role in maintaining reproductive longevity. Furthermore, 14(15)-DiHET, which confers anti-inflammatory effects in cellular senescence, and 9,10-DiHOME, which is negatively associated with obesity, were significantly higher in HL ovaries than in LL ovaries. In contrast, levels of pro-inflammatory metabolites such as 19(S)-HETE and 20-HETE were significantly reduced in HL ovaries compared with LL ovaries.

FOXM1 is a ubiquitously expressed transcription factor that regulates cell proliferation [41,42,43]. Overexpression of FOXM1 has been shown to enhance the proliferative capacity of fibroblasts from 80-year-old individuals and patients with premature aging, thereby delaying cellular senescence [16]. Conversely, inhibition of FOXM1 expression exacerbates aging phenotypes in mice [18]. In this study, FOXM1 expression in the ovaries of 72-week-old ducks in the HL group was significantly lower than in the LL group. The protective effect of FOXM1 against cellular senescence may arise indirectly from reduced DNA damage [44]. Furthermore, FOXM1 overexpression has been reported to alleviate H_2_O_2_-induced premature senescence in mouse fibroblasts [45]. As oxidative stress is a major driver of cellular senescence, FOXM1, an essential regulator of oxidative stress responses and a central participant in tumorigenesis, has become a key focus of research on aging and premature aging. Regarding the effect of H_2_O_2_ on FOXM1 expression, some studies have shown that H_2_O_2_ markedly upregulates its expression [46], whereas other reports indicate significant downregulation [47]. In this study, FOXM1 expression was also significantly decreased in H_2_O_2_-induced oxidative stress models of duck ovarian GCs and KGN cells. It is worth noting that while the protective trend was consistent, the magnitude of the response was more pronounced in the primary duck GCs than in the KGN cell line. Such quantitative differences are likely attributable to the inherent physiological distinctions between a primary, species-specific culture and a transformed, immortalized human cell line. These results indicate that 5-HEPE can effectively alleviate the inhibitory effect of H_2_O_2_ on FOXM1 expression. This suggests that 5-HEPE can improve granulosa cell senescence via FOXM1, which may help extend the laying period of laying ducks.

Although oxylipin levels are strongly associated with ovarian diseases, targeted regulation of the oxylipin pathway remains challenging in therapeutic management. This difficulty arises from the highly complex nature of oxylipin biosynthesis and metabolism. Additionally, the wide structural diversity of oxylipins, coupled with their pleiotropic roles in inflammatory responses, immune regulation, and cellular signaling, complicates the development of precise therapeutic strategies. This study also has several limitations: first, this study has only investigated a single oxylipin; and second, knockdown or overexpression of FOXM1 was not performed in laying ducks to further elucidate its role in ovarian senescence.

## 5. Conclusions

This study is the first to characterize the oxylipin composition of ovaries and plasma in laying ducks in the late laying period. The findings demonstrate that both the types and levels of oxylipins are strongly correlated with ovarian function, and FOXM1 is a key molecular player in this process, suggesting a protective axis where 5-HEPE preserves ovarian function by improving the oxidative stress-induced suppression of FOXM1.

## Figures and Tables

**Table 1:** Primers sequences for qRT-PCR.

Target Genes	Accession Number	Primer Sequences (5′–3′)	
Human			
FOXM1	NM_001243088	F: GCAGCAGAAACGACCGAATC	R: GGTCTTGGGGTGGGAGATTG
GPX4	NM_001039847	F: GAAGATCCAACCCAAGGGCA	R: GACGGTGTCCAAACTTGGTG
β-actin	NM_001101	F: AGACCTGTACGCCAACACAG	R: CCTCGGCCACATTGTGAACT
Duck			
FOXM1	XM_072043035	F: TGTTTAAGCAGCAAAAGCGACA	R: GGGGACAACCAGTCATCCAG
GPX4	XM_038169022	F: GTAGACCTGCTCATCTCGCC	R: GGCTTGAAGACAGACCGTCA
β-actin	NM_001310421	F: CGGACTGTTACCAACACCCA	R: GCCTTCACAGAGGCGAGTAA

## Data Availability

All raw sequencing datasets described in this manuscript have been archived in the Genomic Sequence Archive (GSA) hosted by the China National Center for Bioinformation and Beijing Institute of Genomics, Chinese Academy of Sciences, under the accession number CRA029938. These datasets are publicly retrievable through the official portal at https://ngdc.cncb.ac.cn/gsa, accessed on 25 September 2025.

## Supplementary Materials

The following supporting information can be downloaded at: https://www.mdpi.com/article/10.3390/antiox14121425/s1. Table S1: Egg production records.
